# A Clinical Report on Gude’s Pepto-Mangan

**Published:** 1897-10

**Authors:** A. H. Roler

**Affiliations:** Late Resident Physician Mercy Hospital, Chicago


### A CLINICAL REPORT ON GUDE'S PEPTO-MANGAN.

BY A. H. ROLER, M. D.

Late Resident Physician Mercy Hospital, Chicago.

Iron is an essential part of the human blood in the proportion of 1 part of iron to 230 of red corpuscles. Therefore it is a good example of restoratives, and being found in nearly all foods of man in varying quantity, it necessarily follows that in normal, healthy conditions of the body enough iron is taken into the system in the daily food to supply all the necessary physiological needs of the economy. Iron acts, first, by increasing the oxygen-carrying powers of the blood; second, it converts oxygen in the tissues into ozone; third, it is a general and local astringent.

On account of the difference in their properties or mode of action some preparations of iron have the preference over others. Again, in certain diseases the proportion of iron to the red corpuscles varies so much that we aim to give that preparation which will most nearly meet the requirements.

Manganese, another probable element of the blood, is frequently administered as an adjuvant to iron, and a combination of these two remedies, introduced to the medical profession by Dr. Gude, of Leipsig, and known as Gude's Pepto-Mangan, forms the basis of this clinical report. Success in treating cases where iron is indicated is not always obtained, and for obvious reasons, *e.g.*, a weak or irritable digestive tract which will not tolerate iron and which in itself is an indication. To meet the requirements in such cases one naturally seeks that preparation which will accomplish the desired result with the least untoward effect. In Gude's Pepto-Mangan we seem to have such an article. It is a neutral solution of iron and manganese which is easily assimilated, does not blacken the teeth, does not disturb the digestive system, produces no constipation, acts upon the liver and increases the appetite. These qualities being found in the preparation would indicate that it is one of the best suited for those cases in which iron is indicated. Some of the conditions in which it has been proven to act with beneficial results are anæmia from any cause, chlorosis, dysmenorrhea, amenorrhea, constipation, general debility, etc. A report of a few cases in which this preparation has been used is added.

CASE 1. Mary G., a young married woman, 30 years old, was delivered of twins after prolonged labor followed by an unusual loss of blood. Her condition was profoundly anæmic with anorexia. She was given Gude's Pepto-Mangan in teaspoonful doses in milk, four times daily. Her condition improved steadily, and four weeks after her confinement her color had returned, appetite was much improved, and ill effects of the hemorrhage had almost entirely disappeared.

Case 2. General anæmia in a young girl, Mary A., age 17. She had pale, flabby, mucous surfaces, almost constant headache, constipation, lassitude, difficult and painful menstruation, and she frequently refused food. She was placed upon Gude's Pepto-Mangan, teaspoonful doses three times daily. After five weeks' treatment she has resumed her work as student in the high school, so much improved that further treatment was not thought necessary. Blood count at beginning of treatment was 3,000,000 corpuscles to 1 c. c., and at the end of the fifth week 4,200,000 to 1 c. c.

Case 3. Anna S., age 19, single, by occupation a typewriter. She has suffered for the past year with dysmenorrhea, insomnia, constipation, anorexia. Was generally debilitated. Blood count showed 2,600,000 red discs to 1 c. c. Gude's Pepto-Mangan in drachm doses t. i. d. was given. At the end of six weeks the blood corpuscles had increased to 4,200,000 in 1 c. c. Appetite improved. She can now sleep without any difficulty. Skin has assumed a healthy hue. Dysmenorrhea has not disappeared, but this can be accounted for by a retroverted uterus.

Case 4. Small, puny, delicate child, Dora C., age 5, showed signs of chorea since her fourth year. Gude's Pepto-Mangan has decidedly improved her condition. She can now carry objects with scarcely any tremor and is able to sleep well, and on awakening is not agitated, as she formerly was.

Case 5. Bertha C., age 15, slender, pale, and poorly nourished. Employed in a chewing-gum factory. She has menstruated but twice in the past year, is troubled with palpitation of the heart, obstinate constipation, regurgitation of food at times, tires easily upon slight exertion. She was taken from her work and allowed to do nothing, was given Gude's Pepto-Mangan, dram doses t. i. d. for five weeks. Her general condition improved so much that she was allowed to resume her work. The constipation is now gradually leaving her, and at the time of writing she has gained two pounds. She says she feels better than at any time for a year past. She has menstruated once since treatment was begun, without pain or difficulty of any kind. Blood count at time of beginning treatment 2,700,000 in 1 c. c. A last count, fifth week, 3,300,000 in 1 c. c.—*From The Chicago Medical Recorder, June, 1897.*

